# A bibliometric analysis of metonymy in SSCI-indexed research (2000–2023): retrospect and prospect

**DOI:** 10.3389/fpsyg.2025.1499563

**Published:** 2025-01-27

**Authors:** Yi Sun, Mocheng Lin

**Affiliations:** Fuzhou University of International Studies and Trade, Fuzhou, Fujian, China

**Keywords:** metonymy, bibliometric analysis, Python, metaphor, figurative language

## Abstract

**Introduction:**

Metonymy has gained increasing attention for its role in shaping language, thought, and communication. Despite its prominence, the thematic evolution and future directions of metonymy research remain underexplored. This study seeks to address this gap by analyzing metonymy research published between 2000 and 2023, providing a comprehensive overview of its key trends and emerging themes.

**Methods:**

A bibliometric analysis was conducted using data sourced from the Social Science Citation Index (SSCI) within the Web of Science Core Collection. Co-citation and co-word analysis were employed alongside k-means clustering techniques to identify research themes. Predictive modeling, including ARIMA and LSTM approaches, was used to forecast future research topics based on keyword trends.

**Results:**

The analysis identified 11 key research clusters, highlighting the central role of cognitive and conceptual linguistics in metonymy research, along with its applications in semantics, pragmatics, and multimodal contexts. Predictive modeling suggested the emergence of seven new research themes for 2024–2028, including the interaction between metonymy and discourse, its role in multimodal communication, and its application in social and cultural narratives.

**Discussion:**

This study underscores the interdisciplinary nature of metonymy research, bridging linguistic, cognitive, and social dimensions. The findings highlight promising areas for future exploration, namely, its integration into digital communication and its impact on cultural identity construction. The methodological approach offers a robust framework for analyzing and predicting research trends, paving the way for innovative contributions to the field.

## 1 Introduction

Conceptual Metaphor Theory (CMT), pioneered by Lakoff and Johnson (1980), heralded a new chapter in the exploration of the essence of metaphors. This theory posits that metaphors are not merely linguistic embellishments but are deeply ingrained in everyday language use. Originating from inquiries into the cognitive foundations of language practice, CMT research has progressively permeated various interdisciplinary fields, encompassing metaphor and culture (Kövecses, 2005, 2006), metaphor and communicative discourse (Zinken and Mussolf, 2009), and the neuroscientific foundations of metaphor (Feldman and Narayanan, 2004; Grady and Ascoli, 2017).

While metaphors have garnered significant scholarly attention, an equally important cognitive-linguistic phenomenon, metonymy, has not been explored as extensively. Metonymy, often studied alongside metaphor, is part of the everyday way of thinking, grounded in experience and governed by systematic principles that structure our thought and actions (Gibbs, 1994, pp. 324–333). The precise definition of metonymy has been a topic of scholarly debate, with two main perspectives emerging. The first considers metonymy as an intra-domain conceptual mapping (Lakoff and Johnson, 1980), involving representational relationships within a single conceptual domain. For example, in the sentence “The guitar has been drinking heavily,” the guitar stands for the guitarist. The second perspective views metonymy as a “reference point” phenomenon (Langacker, 1993; Kövecses and Radden, 1998), as in “He has a Picasso,” where the artist serves as a reference point for his artwork. Panther and Radden (1999) pointed out that “metonymy is a cognitive phenomenon that may be even more fundamental than metaphor” (p. 1). Subsequent examinations by scholars like Barcelona (2003/2000), Dirven and Pörings (2002), Panther and Tornburg (2003), and Panther et al. (2009) have confirmed Panther and Radden's (1999) speculation. Based on this, research has extended to various facets of metonymy, including its cognitive operations (El Yamlahi and Cortés de los Ríos, 2022), pragmatic functions (Pannain, 2017), and its intersection with other linguistic phenomena (Yurchenko et al., 2020). A landmark contribution is Littlemore's (2015) comprehensive overview metonymy research, emphasizing its significance in cognitive and discourse studies and highlighting its pervasive influence in language and communication.

Despite the valuable insights provided by these studies, there remains a conspicuous gap in the literature regarding a holistic analysis of the thematic evolution and future trends of metonymy research. To address this gap, this study aims to systematically review and analyze metonymy research from 2000 to 2023, using data sourced from the Social Science Citation Index (SSCI) within the Web of Science Core Collection. SSCI offers a more targeted selection of journals with a robust focus on social science disciplines, ensuring that the literature surveyed in this study represents the most relevant and influential contributions to the cognitive and linguistic dimensions of metonymy research. By employing advanced bibliometric and time series analysis techniques, this study seeks to provide a comprehensive overview of metonymy research, tracing its development over the past two decades and identifying emerging trends and future directions. Specifically, this study aims to answer the following research questions:

1. What have been the research focuses on metonymy over the past two decades?
2. What are the prospective research topics for the future development of metonymy research?
3. What are the evolutionary trends in metonymy research?

## 2 Methodology

Bibliometric analysis is a well-established quantitative method in academic research that facilitates the systematic assessment of scholarly literature. This method employs a variety of techniques, including citation analysis, co-citation analysis, and keyword co-occurrence analysis, to uncover patterns, trends, and relationships within a given corpus of literature. These methodologies enable researchers to delineate the intellectual structure of a field, monitor its development over time, and forecast potential future research trajectories. By examining publication patterns, citation networks, and co-authorship relationships, bibliometric methods offer quantitative insights into research trends, key contributors, and thematic evolutions (Börner et al., 2003). The versatility of bibliometric approaches allows them to encompass a wide array of scholarly disciplines, providing both micro and macro-level perspectives (Van Raan, 2005; Xiao and Li, 2021). Additionally, bibliometrics provides a statistical means to evaluate and quantify research output and growth trends in specific academic fields (Chen et al., 2021).

The present study aims to forecast the evolution of metonymy research topics by analyzing the relationship between topics and keywords. Established research topics are typically characterized by specific combinations of multiple keywords, and shifts in these combinations often signal the emergence and development of new research areas. As topics evolve, new keyword combinations emerge, driving recurring cycles of conceptual deconstruction and reconstruction. This dynamic relationship between research topics and keywords is observable across different academic fields (Liang et al., 2023). The literature on a specific research topic, once organized and processed, forms a topic-keyword representation that conveys its core essence. By selecting and clustering keywords, researchers can abstract a set of keywords to form keyword groups that, in conjunction with word frequency analysis, represent the research topic. Throughout this process, research topics exhibit continuity, evolving from original topics to new topics through the reorganization and reinterpretation of keyword groups. Consequently, changes in keyword combinations can reflect both the evolution of existing topics and the emergence of new ones.

Furthermore, the development of research topics typically follows a predictable life cycle, comprising stages such as emergence, growth, maturity, stabilization, and decline. Topics do not appear or disappear abruptly; instead, their developmental trajectories are often traceable. Therefore, short-term predictive analysis of topic trends using time series data is both feasible and valuable. Based on this understanding, the study hypothesizes that the temporal evolution of metonymy research topics is continuous and influenced by preceding stages. This continuity suggests that it is possible to construct time series models based on historical data to predict future trends in metonymy research.

**Figure 1:**
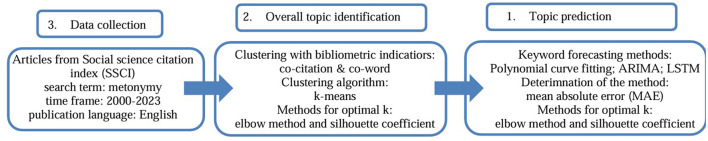
Research procedure.

As illustrated in Figure 1, the methodology for predicting research trends and topic evolution in metonymy research involves three primary steps: data collection, identifying overarching topics, and forecasting future topic trend. This approach begins by gathering relevant research articles, then identifies common themes and patterns based on keywords and citations, and finally uses historical data to anticipate future developments in these themes.

Each step is detailed as follows:

### 2.1 Data collection

For the systematic review of metonymy research, data were sourced from the Social Science Citation Index (SSCI) within the Web of Science (WOS) Core Collection, provided by Clarivate Analytics. The Web of Science repository offers access to high-impact publications and their citation data across the natural and social sciences. Utilizing the Core Collection ensured the inclusion of high-quality materials and detailed citation data. The systematic literature retrieval strategy was as follows:

1. “Metonymy” was selected as the core search term, with a temporal boundary set from 1 January 2000 to 31 December 2023.
2. The search was restricted to articles, and only publications in English were considered to ensure consistency and comparability in the analysis.
3. After the initial online retrieval, a manual screening process was conducted to exclude publications that were unrelated to metonymy or lacked keywords.

**Figure 2:**
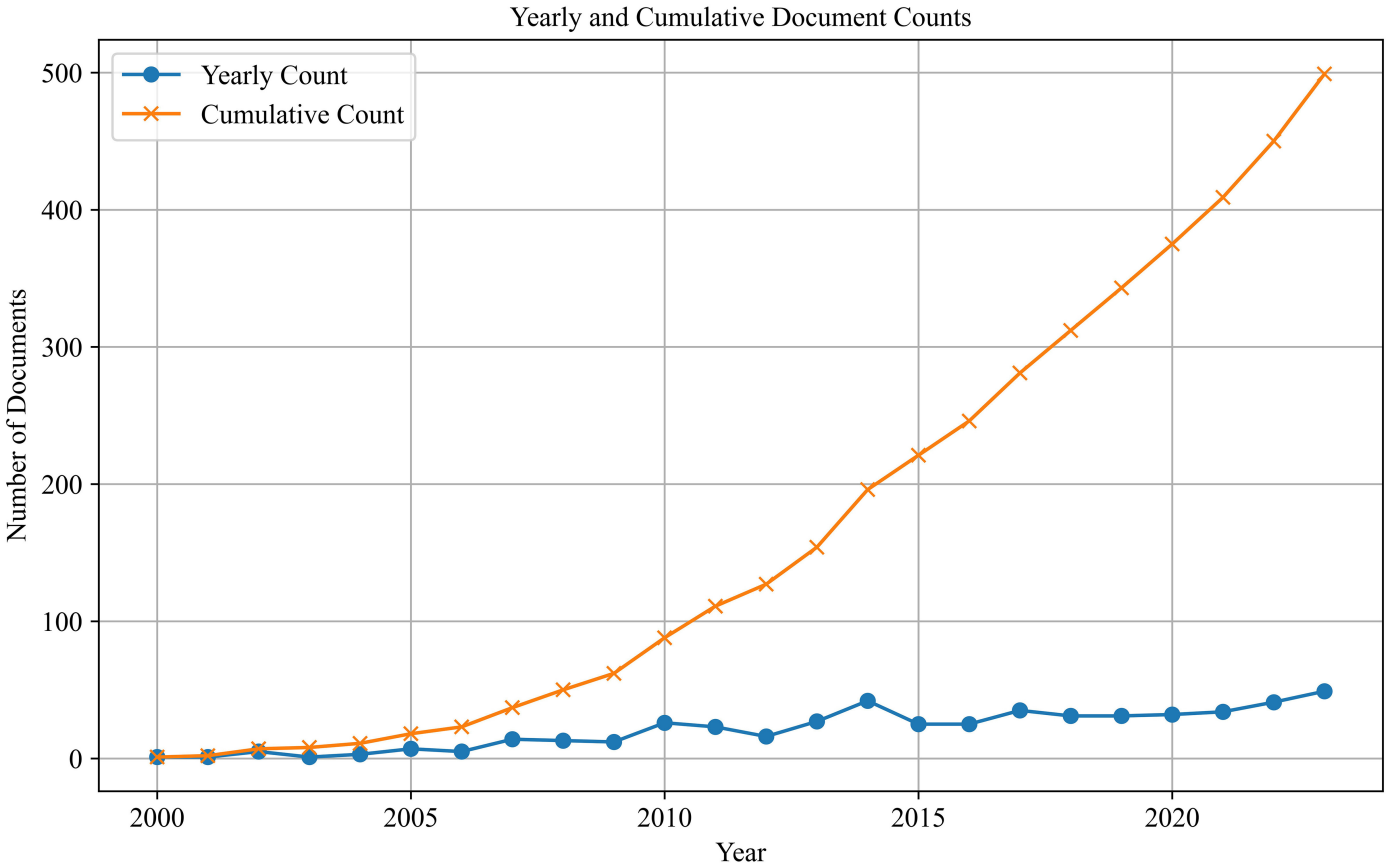
Yearly and cumulative article counts.

The initial search resulted in 589 publications. After the filtering process, a final total of 499 publications were included in the dataset for subsequent analysis. The distribution of publications over time is depicted in Figure 2, which shows a steady increase in the number of publications, with an acceleration in cumulative counts over time. This trend suggests that the field of metonymy has transitioned from its early stages into a mature phase characterized by rapid growth and increased scholarly attention.

### 2.2 Overall topic identification

Co-citation analysis and co-word analysis are two fundamental approaches in bibliometric research that provide complementary perspectives for understanding the structure and development of scientific fields. Co-citation analysis, developed from Kessler's (1963) concept of bibliographic coupling and formalized by Small (1973), identifies relationships between documents based on their shared references in a third document. Despite some criticism, Small (1974) argued that co-citation patterns, particularly multiple citation connections, are significant indicators of research specialties and disciplines (Small and Greenlee, 1980; Small and Crane, 1979). At a higher level of abstraction, Price (1965) utilized ISI data to theorize about the structure of science itself, exploring networks of scientific papers to identify research fronts. Cozzens (1985) further observed that co-citation studies appear to confirm Price's (1970) hypotheses regarding significant intellectual focus areas, as evidenced by referencing patterns within active specialty groups. In the study of research specialties, various works have explored reference networks (Baldi and Hargens, 1997; Price, 1965), the codification and accumulation of knowledge in various fields (Cozzens, 1985; Lewis, 1980), and the use of journal-to-journal citation data to identify the emergence and transformation of specialties (Van den Besselaar and Leydesdorff, 1996). Co-word analysis, introduced by Callon et al. (1983), maps the co-occurrence of specific terms across documents, revealing how concepts cluster together within and across fields. Co-word analysis has been applied to study various fields, including biotechnology, artificial intelligence, cancer research, polymer chemistry, and acidification research (Rip and Courtial, 1984; Courtial and Law, 1989; Oehler et al., 1989; Callon et al., 1991; Law and Whitaker, 1992; Courtial, 1994; Ding et al., 2001; Coulter et al., 1998). While co-word analysis has faced criticism due to the evolving nature of language (Leydesdorff, 1997), it remains a powerful tool for tracking scientific change and development (Courtial, 1998).

To maximize the insights from both approaches, clustering methods were developed to integrate co-citation and co-word data. Clustering approaches, widely used in bibliometric research, are often designed to address specific needs, making the adaptation of generic clustering techniques to different tasks complex (Jain et al., 1999). Consequently, clustering techniques, such as k-means, hierarchical clustering, and topic models, leverage indicators like word co-occurrence, co-citation patterns, and bibliographic coupling to group related research topics (Zhang et al., 2017; Funk and Owen-Smith, 2016; Li et al., 2014; Zhao and Strotmann, 2014). Various combinations of clustering algorithms and bibliometric indicators have been evaluated across multiple datasets and tasks. For example, Boyack et al. (2011) assessed the accuracy of five clustering approaches on biomedical articles from Medline; Ding and Chen (2014) compared the effectiveness of topic models, co-word analysis, and co-citation analysis for topic detection and tracking; Zhang et al. (2016) explored the usefulness of k-means, hierarchical clustering, and topic models in analyzing academic proposals granted by the National Science Foundation; Klavans and Boyack (2017) tested the ability of directional citations, bibliographic couplings, and co-citations to accurately represent scientific and technical knowledge taxonomies. K-means, a widely used clustering method, remains popular due to its simplicity and low computational complexity, despite being one of the oldest clustering methods (Jain, 2010).

In the context of this study, we utilized Python to conduct a comprehensive topic identification process. The analysis began with constructing two essential matrices: a co-citation matrix and a TF-IDF keyword matrix. These matrices formed the foundation for the subsequent clustering process using the k-means algorithm.

The first step involves preprocessing the keywords extracted from research articles. We used NLTK (nltk) for preprocessing, including converting keywords to lowercase and applying lemmatization via the WordNetLemmatizer to standardize different word forms. Keywords appearing fewer than three times were filtered out using Pandas (pandas) to retain only the most relevant terms. Simultaneously, we constructed a co-citation matrix by creating a network graph using NetworkX (networkx), where nodes represent references, and edges represent the co-citation relationships between articles, indicating the strength of the relationship. For keyword analysis, we generated a TF-IDF matrix using Scikit-learn's (sklearn) TfidfVectorizer. This matrix captured the importance of each keyword by adjusting for its frequency within the entire dataset.

The second step involves the integration of co-citation and keyword information. Both matrices were standardized to ensure their equal contribution to the clustering process. This is accomplished using Scikit-learn's StandardScaler. The standardized co-citation matrix and TF-IDF matrix were then combined into a single feature matrix that integrates both citation relationships and semantic content. This combined matrix offered a comprehensive representation of each document, capturing content and citation-based similarities.

Third, to prepare the data for clustering, we applied t-distributed Stochastic Neighbor Embedding (t-SNE) for dimensionality reduction, using TSNE from Scikit-learn. This technique reduced the complexity of the combined feature matrix, making it easier to visualize the clusters in a two-dimensional space. Next, we determined the optimal number of clusters using two approaches:

1. The Elbow method, which is implemented to calculate the Sum of Squared Errors (SSE) for different numbers of clusters.
2. Silhouette analysis, using silhouette_score from Scikit-learn, to measure the quality of clustering.

Once the optimal number of clusters is identified, we use the k-means algorithm to partition the dataset.

Finally, we conducted an analysis of each cluster to identify the top keywords that characterize it. To achieve this, we created a topic-keyword probability matrix, which was constructed using the clustered keyword data. This matrix quantified the likelihood of each keyword occurring within a cluster and is normalized across the dataset to provide accurate comparisons. The top 20 keywords for each cluster were extracted based on their occurrence probabilities, offering a refined view of the most significant terms associated with each topic. The Matplotlib (matplotlib.pyplot) and Seaborn (seaborn) libraries were used to generate bar plots and visualizations, which depict:

1. The distribution of documents across clusters.
2. The frequency of the top keywords within each cluster.

### 2.3 Topic prediction

The evolution of research topics typically follows a pattern of continuity, with keyword trends reflecting underlying inertia. Keyword frequency serves as one of the most direct and effective external indicators of a topic's state. Compared to manually set indicators for predicting topic states, raw word frequency indicators are inherently more objective and accurate, boasting strong scientific validity and broad applicability. In predictive research, these indicators help minimize errors, thereby enhancing the reliability of the predictions. Accordingly, this study employed time series analysis to forecast future keyword frequencies, which were subsequently used to calculate vector adjustment coefficients. To comprehensively and accurately capture the trends in word frequency evolution, a time window of 1 year was employed for multi-step forecasting.

Three forecasting methods were utilized to predict keyword frequencies: polynomial curve fitting, ARIMA modeling, and LSTM modeling. The study adopted a recursive prediction approach, wherein the frequency of keywords for year n+1 is predicted based on the data from period (2000 to year n). The predicted value for year n+1 was then integrated back into the original dataset, which was subsequently used to predict the data for year n+2, and so forth.

To minimize prediction errors, the mean absolute error (MAE) was employed as the error evaluation metric, determining the final prediction model and forecasting method. Following this, the k-means algorithm, implemented in Python, was applied once again to cluster high-frequency keywords, thereby identifying the predicted topics. This approach, compared to qualitative methods, is more scientific and better reflects the temporal trends and inertia of topics, with Python's capabilities ensuring robust and reproducible results.

We began by constructing a Keyword-Year Frequency Matrix, using Pandas (pandas) to extract keyword frequencies for each year in our dataset (2000–2023). Missing values were handled using linear interpolation from Pandas, ensuring continuity across the years. After filtering out less significant keywords based on a predefined minimum occurrence threshold, the dataset was standardized using StandardScaler from Scikit-learn (sklearn). This step transformed the data to have a mean of zero and a standard deviation of one, facilitating accurate modeling by eliminating bias from differing scales. Following preprocessing, the keyword frequency data was standardized using Python's StandardScaler. This standardization transformed the data to have a mean of zero and a standard deviation of one, facilitating effective modeling and ensuring that all variables contribute equally to the prediction process.

The next phase involved the use of three distinct recursive prediction models to forecast future keyword frequencies:

1. Polynomial Recursive Model: This involved fitting polynomial regression models of varying degrees to historical keyword data using Python's scikit-learn library. The model with the highest *R*^2^ value was selected, and future keyword frequencies are recursively predicted.
2. ARIMA Recursive Model: Python's pmdarima library was used to automatically select the best-fit ARIMA model parameters via the auto_arima function. The chosen ARIMA model was then used to recursively forecast keyword frequencies, ensuring non-negative predictions.
3. LSTM Recursive Model: Data was reshaped to fit the input requirements of LSTM models using Python's numpy and keras libraries. Hyperparameters for the LSTM model, such as the number of units and learning rate, were optimized using a Random Search within the Keras Tuner framework. The best-tuned LSTM model was then used to recursively predict future keyword frequencies.

These predictive models were evaluated based on their Mean Absolute Error (MAE) over the predicted periods. For each model, Python's scikit-learn library is used to compute the MAE for each future year to assess prediction accuracy. The predicted frequencies were then converted back to their original scale using inverse standardization, ensuring comparability with the original data.

Finally, the k-means algorithm were applied to cluster the predicted keywords and identify distinct research themes. This approach ensured a robust and scientifically valid method of forecasting keyword trends, providing valuable insights into the future trajectory of research topics in academic fields.

## 3 Results

### 3.1 Clustering metonymy research topics using co-citation and co-word analysis

**Figure 3:**
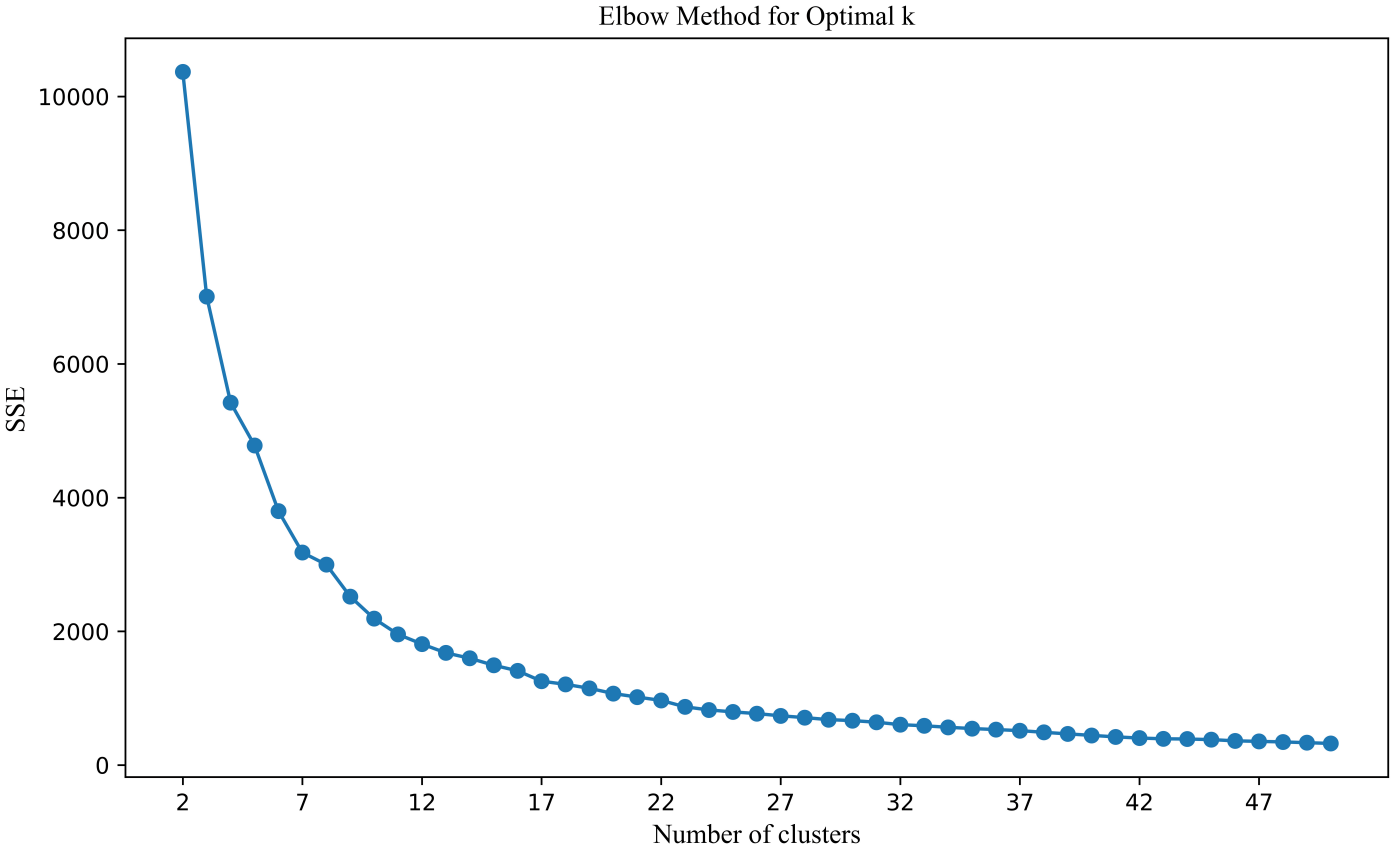
Elbow method for optimal K.

**Figure 4:**
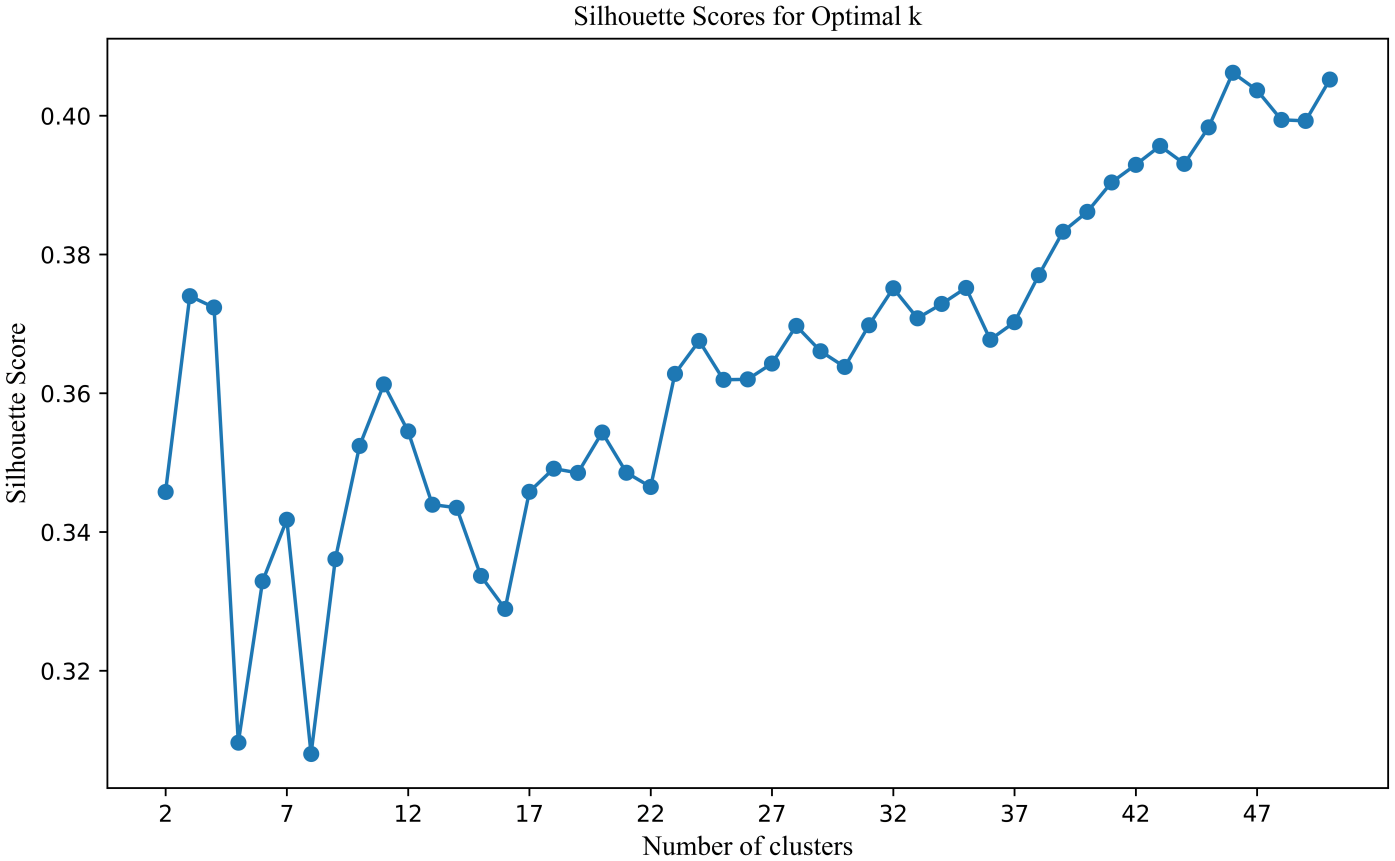
Silhouette scores for optimal K.

By combining co-citation and co-word analysis with the k-means algorithm, clusters were formed, and keywords for each cluster were extracted. The optimal number of clusters (k) was determined using the elbow method and silhouette scores, as shown in Figure 3. This method plots the Sum of Squared Errors (SSE) against the number of clusters. The plot reveals a noticeable “elbow” point at *k* = 11, where the rate of decrease in SSE significantly slows down. This inflection point suggests that 11 clusters strike a balance between underfitting and overfitting, providing a meaningful partitioning of the data. Silhouette analysis, depicted in Figure 4, further validated the choice of the optimal number of clusters. The silhouette score evaluates the quality of clustering by measuring how similar each point is to others within its cluster compared to points in other clusters. Higher silhouette scores indicate better-defined clusters. The analysis showed that *k* = 11 yielded a relatively high silhouette score, supporting the choice of 11 clusters for subsequent analysis.

**Figure 5:**
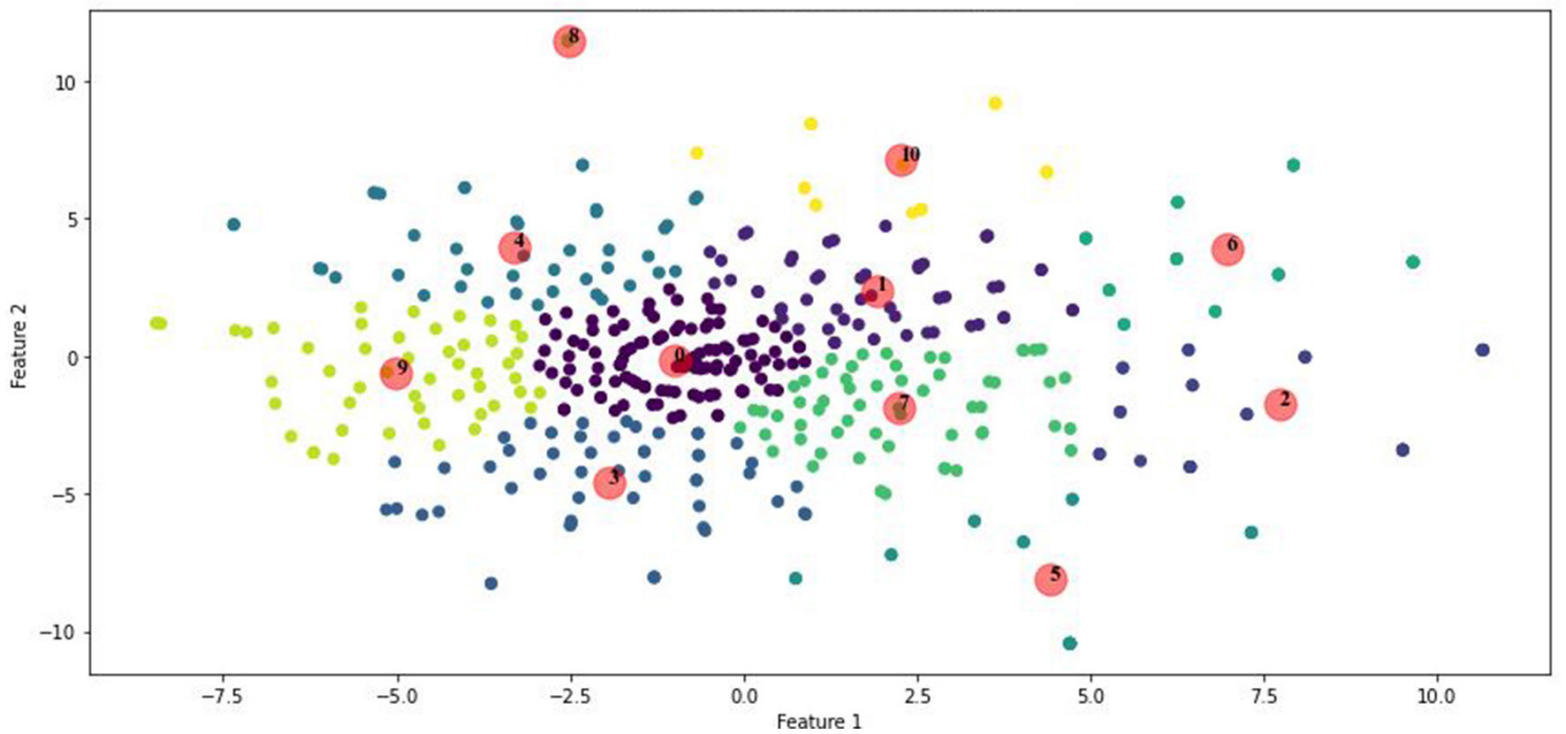
K-means clustering with optimal K.

The clustering result with *k* = 11 was visualized, as shown in Figure 5. The plot presents the clusters in a two-dimensional space, with each color representing a different cluster and centroids marked with red circles. This visualization confirms the separation and cohesion of the clusters, indicating distinct research topics within the metonymy studies.

To ensure topic completeness and interpretability, the top 20 words with the highest probability distribution in each scientific topic were extracted and listed in descending order of frequency within each cluster, as shown in Table 1, to represent the topic content. This table presents the detailed keyword analysis and article distribution of each cluster in metonymy research. Thus, the research topics and topic-keyword sets in the field of “metonymy” from 2000 to 2023 were obtained. The top keywords reflect the primary research interests and thematic focus within each cluster, demonstrating the diversity and specific areas of study in metonymy research. The identified topics encapsulate diverse areas of focus in metonymy research, characterized by shared keywords and co-citation patterns. These clusters provide a structured overview of the research landscape, highlighting the evolution and differentiation of topics within metonymy studies over the specified period.

**Table 1:** Summary of keywords of clusters.

**Cluster-ID**	**Size**	**Top keywords**
0	128	metonymy; metaphor; cognitive linguistics; conceptual metonymy; emotion; semantic change; syntax; visual metaphor; embodiment; figurative language; construction; rhetoric; irony; polysemy; gender; adjective; conceptual integration; visual metonymy; experimental pragmatic; n400
1	54	metonymy; metaphor; conceptual metaphor; cognitive linguistics; conceptual metonymy; pragmatic; figurative language; language; polysemy; visual metonymy; visual metaphor; conceptualization; cultural model; cognitive operation; autism; emotion; English; euphemism; conceptual metaphor theory; development
2	31	metonymy; polysemy; metaphor; homonymy; lexical ambiguity; construction; conceptual metaphor; pragmatic; idiom; metaphor and metonymy; construal; n400; blending; cognitive operation; speech act; multimodality; conceptual metonymy; euphemism; semiotics; high-level metonymy
3	52	metonymy; metaphor; semantics; corpus linguistics; discourse; pragmatic; iconicity; cognitive linguistics; lexical semantics; conceptual metaphor theory; conceptual metonymy; cognitive grammar; idiom; figurative language; coercion; conceptual metaphor; motivation; construal; construction; construction grammar
4	43	metonymy; metaphor; emotion; figurative language; indexicality; iconicity; semantics; conceptual metaphor; coercion; frame; motivation; conceptual metonymy; visual metonymy; cognitive grammar; contiguity; adjective; polysemy; cognitive semantics; novel metaphor; blending
5	23	metonymy; metaphor; figurative language; euphemism; persuasion; italian; conceptual blending; cognitive semantics; autism spectrum disorder; iconicity; polysemy; image schema; indexicality; cognitive linguistics; English; high-level metonymy; conceptual metaphor; embodiment; political cartoon; emotion
6	26	metonymy; metaphor; autism spectrum disorder; williams syndrome; figurative language; pragmatic; semantic change; autism; novel metaphor; inference; conceptual metonymy; frame; corpus; language; idiom; image schema; metaphor and metonymy; creativity; word-formation; cognitive linguistics
7	58	metonymy; metaphor; polysemy; figurative language; pragmatic; cognitive linguistics; image schema; creativity; conceptual metonymy; multimodal metaphor; homonymy; English; autism spectrum disorder; emotion; novel metaphor; cognitive model; cognitive semantics; development; semantics; narrative
8	11	metonymy; metaphor; multimodality; picture book; cognitive operation; cognitive linguistics; multimodal metaphor; corpus; creativity; visual metonymy; cognitive model; gender; contiguity; embodiment
9	51	metonymy; metaphor; construction; cognitive linguistics; figurative language; analogy; rhetoric; emotion; trope; Chinese; metaphor and metonymy; conceptual metonymy; conceptual metaphor; creativity; persuasion; discourse; cognitive semantics; compound; frame; logical metonymy
10	22	metonymy; metaphor; visual metaphor; conceptual metaphor; political cartoon; speech act; cognitive linguistics; cognitive semantics; analogy; irony; cultural model; visual metonymy; image schema; multimodality; discourse; motivation; high-level metonymy; corpus linguistics; conceptual metonymy; cognitive model

### 3.2 Metonymy research topic forecast

**Figure 6:**
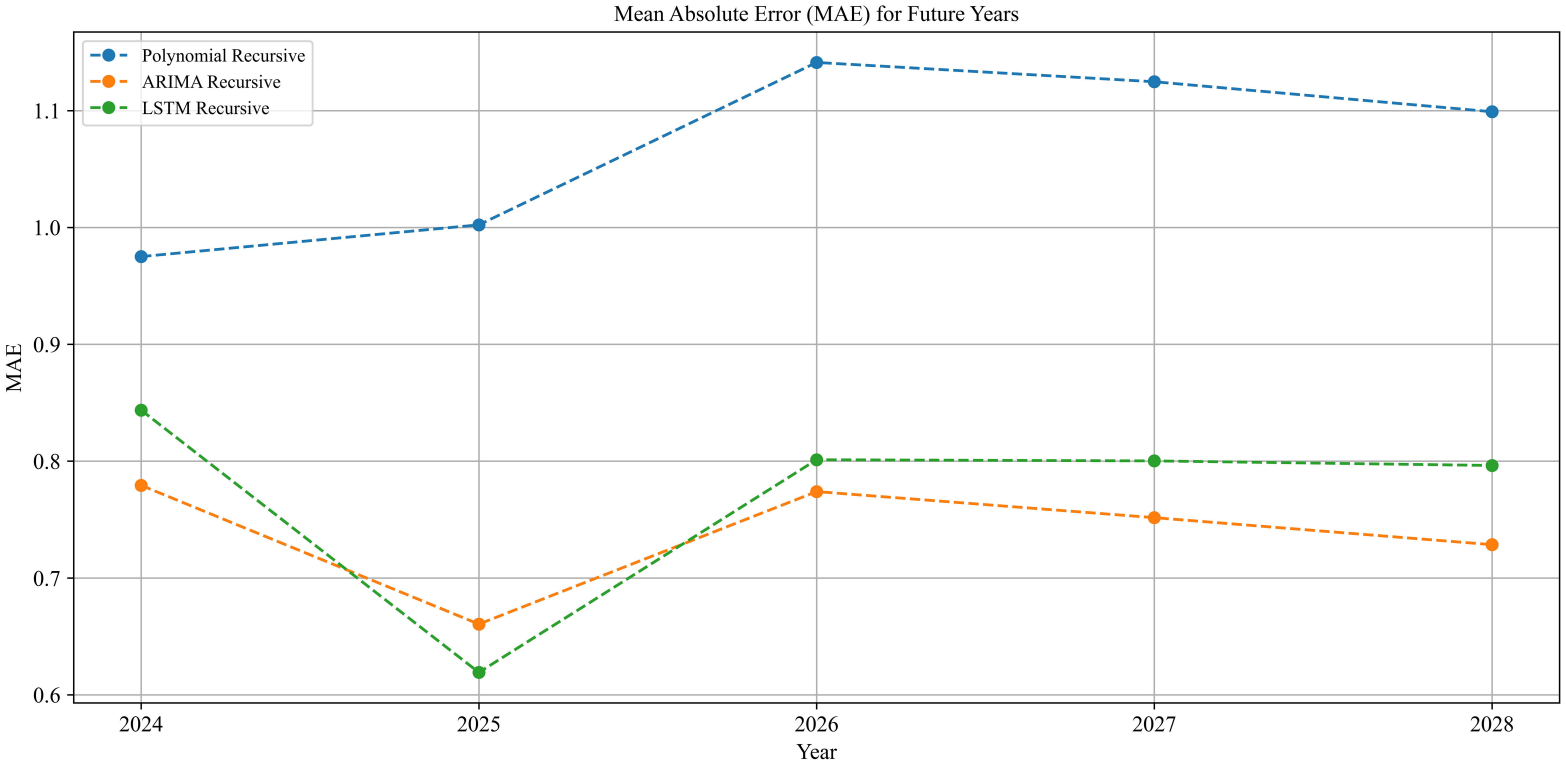
Mean absolute error (MAE) comparison.

Building on the previously generated topic-keyword set, 80 keywords were identified. Among these, 36 keywords (45%) have a length greater than two words, and 6 keywords (7.5%) have a length greater than three words. To determine the most accurate forecasting method, the mean absolute error (MAE) of three methods—polynomial curve fitting, ARIMA, and LSTM—was compared, as illustrated in Figure 6. The results indicate that the polynomial curve fitting method performed worse than both ARIMA and LSTM. Although LSTM and ARIMA exhibited similar predictive performance, the ARIMA model generally yielded a lower MAE, suggesting superior forecasting accuracy. Consequently, the ARIMA model was selected for predicting word frequency trends.

**Figure 7:**
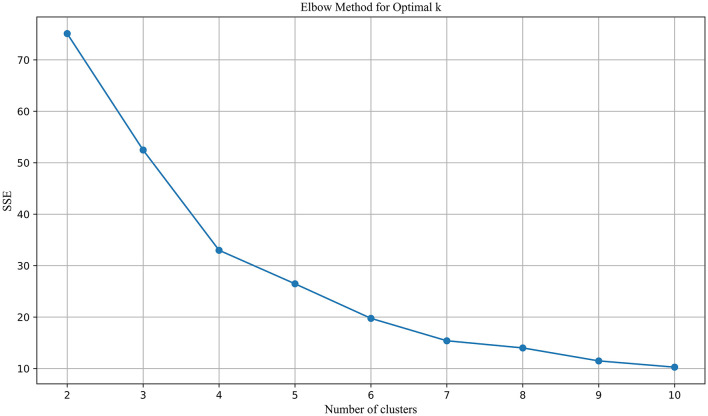
Elbow method for optimal K in PT clustering.

**Figure 8:**
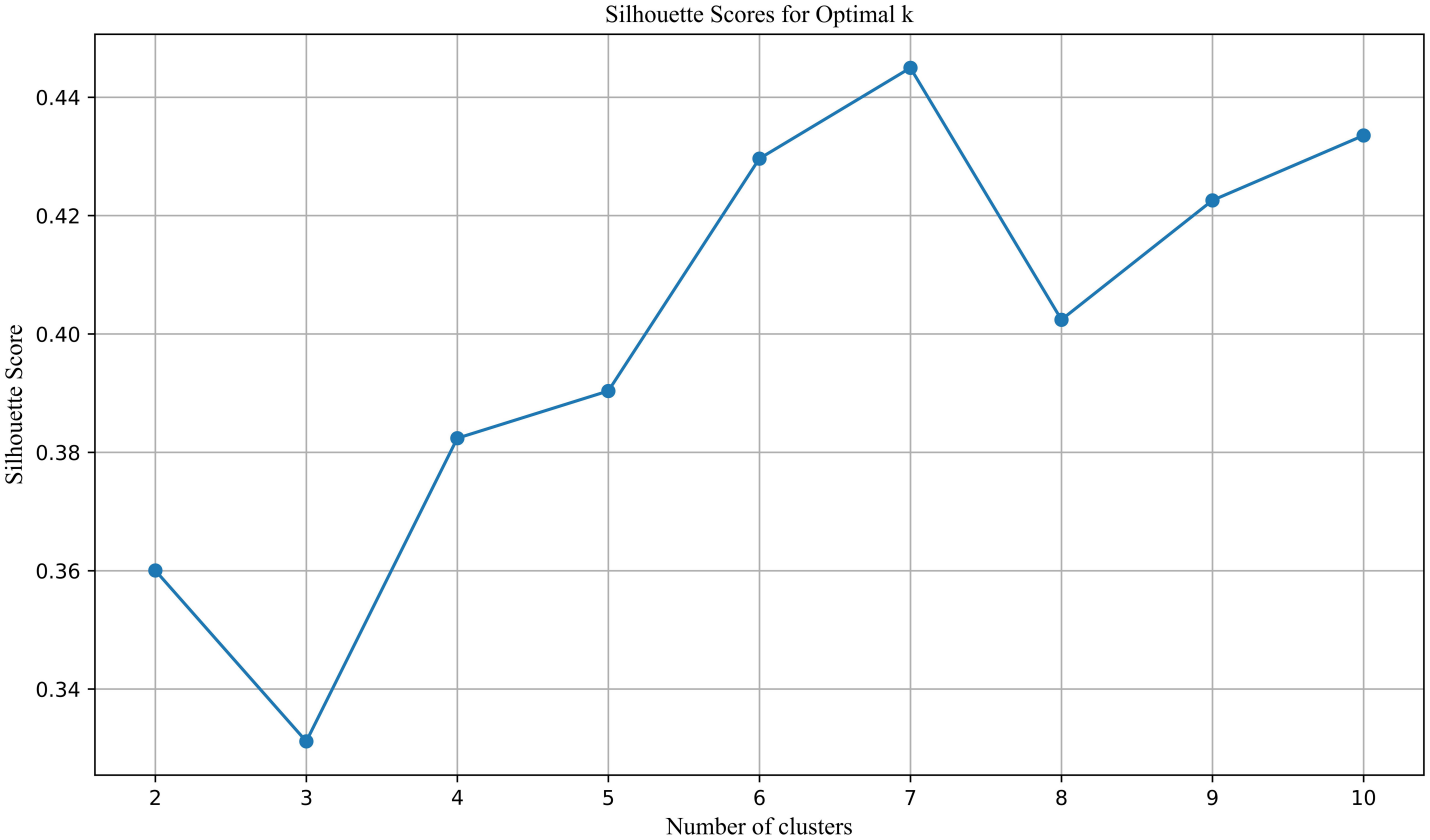
Silhouette scores for optimal K in PT clustering.

Using the ARIMA model, keyword trends for the next 5 years (2024–2028) were forecasted. To further analyze the predicted topics, 51 keywords with an average frequency above the median of the original dataset were selected for clustering. These keywords are presented in descending order of frequency in Table 2. The same clustering methodology as previously described was applied. The optimal number of clusters was determined using a combination of the Elbow method (Figure 7) and the Silhouette coefficient (Figure 8). The analysis revealed that clustering with *k* = 7 provided the highest silhouette coefficient, corroborated by the Elbow method, thus *k* = 7 was chosen for the clustering of predicted topics.

**Table 2:** Summary of forecast keywords.

1	Metonymy	21	Semantics	41	Autism spectrum disorder
2	Metaphor	22	Homonymy	42	Inference
3	Figurative language	23	Corpus linguistics	43	Motivation
4	Conceptual metonymy	24	Cognitive semantics	44	High-level metonymy
5	Cognitive linguistics	25	Advertising	45	Race
6	Persuasion	26	Coercion	46	Conceptual blending
7	Multimodality	27	Compound	47	Metaphor and metonymy
8	Conceptual metaphor	28	Lexical ambiguity	48	Memory
9	Polysemy	29	Semantic change	49	Irony
10	Creativity	30	Rhetoric	50	Logical metonymy
11	Language	31	Gender	51	Multimodal metaphor
12	Visual metonymy	32	Construction		
13	Visual metaphor	33	Conceptualization		
14	Discourse	34	Construal		
15	Development	35	Conceptual integration		
16	Emotion	36	Contiguity		
17	Embodiment	37	Subjectivity		
18	Iconicity	38	Blending		
19	Word-formation	39	Euphemism		
20	Corpus	40	Indexicality		

The clustering results for the predicted topics (PT) are summarized in Table 3, with each cluster characterized by a set of keywords representing anticipated focus areas in metonymy research from 2024 to 2028.

**Table 3:** Summary of keywords of predicted topic.

**PT Cluster ID**	**Keywords**
0	polysemy, homonymy, lexical ambiguity, construction, construal
1	figurative language, language, development, word-formation, advertising, conceptualization, autism spectrum disorder, inference
2	persuasion, blending, euphemism, high-level metonymy, conceptual blending
3	corpus, corpus linguistics, compound, semantic change, rhetoric, gender, conceptual integration, contiguity, subjectivity, race, memory, irony
4	discourse, iconicity, semantics, cognitive semantics, coercion, indexicality, motivation
5	metonymy, conceptual metonymy, cognitive linguistics, visual metonymy, emotion, embodiment, logical metonymy
6	metaphor, multimodality, conceptual metaphor, creativity, visual metaphor, metaphor and metonymy, multimodal metaphor

## 4 Discussion

The above clustering analysis during identified 11 distinct research topics, each representing key areas of interest within metonymy research from 2000 to 2023. In addition, a forecast for the next 5 years (2024–2028) predicts seven emerging topics that are likely to shape the future trajectory of the field. This section discusses the primary focuses of the 11 clusters from 2000 to 2023, the temporal distribution of research activities, and the trends and prospects for future research based on the predicted clusters.

### 4.1 Research focuses on metonymy from 2000 to 2023

The clustering analysis identified 11 distinct research topics within the field of metonymy. Each cluster represents a specific area of focus, reflecting the interdisciplinary approaches and multifaceted nature of metonymy research. These include theoretical constructs, specific genres, investigative domains, and socio-cultural constructs. The shared keywords of these clusters reveal two important trends in the field of metonymy research. First, almost all clusters include keywords related to cognitive linguistics and figurative language, indicating a strong foundation in understanding metonymy through cognitive frameworks. The consistent presence of terms like “metonymy,” “metaphor,” and “cognitive linguistics” underscores the importance of these concepts in metonymy research, highlighting the interplay between metonymy and metaphor. This further confirms our previous claim that these two figurative devices are often studied together. Terms like “conceptual metonymy,” “conceptual metaphor,” and “blending” appear frequently, reflecting ongoing interest in how these mechanisms interact within language and thought. Second, pragmatic and semantic aspects are prominently featured, with keywords such as “pragmatic,” “semantics,” and “lexical semantics” appearing in multiple clusters. This indicates a focus on how metonymy operates at the level of meaning and use in different contexts. The sizes of the clusters vary significantly, with Cluster 0 being the largest (128 articles) and Cluster 8 the smallest (11 articles). This disparity suggests that certain areas, such as cognitive linguistics and conceptual metonymy, have received more extensive research attention compared to more specialized topics like multimodal metonymy. However, specialized topics can also provide several insights into current research focuses of metonymy research.

1) Cognitive and Conceptual Linguistics

A significant portion of metonymy research is anchored in cognitive and conceptual linguistics. This includes studies on how metonymy operates within cognitive frameworks, examining mental processes and conceptual integration. For example, Cluster 0 (Cognitive Linguistics and Conceptual Metonymy) underscores the centrality of cognitive linguistics in metonymy research. This cluster also reflects a broader engagement with metonymy as a cognitive process that intersects with other theoretical constructs and applied domains. Terms like “syntax,” “visual metaphor,” and “embodiment” indicate a strong interest in cognitive and syntactic frameworks, as well as visual and embodied manifestations. The prominence of “experimental pragmatic” and “n400” suggests engagement with experimental and neurocognitive approaches, reflecting a trend toward empirical validation of theoretical constructs.

2) Semantic and Pragmatic Dimensions

Metonymy research extensively covers semantic and pragmatic dimensions, focusing on how metonymic expressions function at the level of meaning and use. Cluster 2 (Polysemy and Lexical Ambiguity) highlights the relationship between metonymy, polysemy, and lexical ambiguity. The focus on terms like “homonymy,” “idiom,” and “blending” suggests an exploration of how metonymy contributes to multiple meanings and complex word forms. Additionally, Cluster 3 (Semantics and Corpus Linguistics) emphasizes detailed examinations of metonymy within language use and structure, integrating metonymic and metaphoric frameworks.

3) Multimodal and Visual Metonymy

The expansion of metonymy research into multimodal and visual genres is a notable trend. Cluster 8 (Multimodal Metonymy in Picture Books) focuses on the use of metonymy in multimodal contexts, particularly in picture books. This cluster suggests an interest in how metonymy functions in multimodal storytelling and educational materials. Similarly, Cluster 10 (Visual Metaphor and Political Cartoons) highlights the intersection of visual metaphor and metonymy in political cartoons and other visual media, focusing on the complex interplay between verbal and visual elements in conveying political and cultural messages.

4) Social and Cultural Applications

Research has increasingly applied metonymy theory to social and cultural contexts, examining its role in shaping societal discourses and identity constructions. Cluster 5 (Persuasion and Political Communication) focuses on the persuasive aspects of figurative language, including metonymy. The inclusion of terms like “euphemism,” “persuasion,” and “political cartoon” points to an interest in the rhetorical uses of metonymy. Meanwhile, Cluster 6 (Metonymy and Neurodiversity) addresses metonymy in relation to autism spectrum disorder and novel metaphor, exploring cognitive differences in neurodiverse populations.

5) Corpus and Data-Driven Approaches

Methodological innovations have played a significant role in advancing metonymy research. Cluster 3 (Semantics and Corpus Linguistics) underscores the use of corpus methodologies to study semantic change and the intersection of metonymy with social issues such as gender and race. This empirical foundation supports more precise and comprehensive analyses of how metonymy functions in natural language use.

6) Emotional and Iconic Dimensions

The emotional and iconic dimensions of metonymy are also prominent research areas. Cluster 4 (Emotional and Iconic Metonymy) centers on the emotional and iconic dimensions of metonymy, highlighting how metonymy conveys emotional states and its relationship with iconic signs. This suggests a nuanced investigation into how metonymy interacts with other semiotic resources to create meaning.

### 4.2 Trends and prospects in metonymy research

The k-means clustering analysis provides valuable insights into the future directions and emerging themes within metonymy research. These predicted topic clusters highlight key areas of focus and potential developments, reflecting the evolving landscape of metonymy studies.

1) Semantic Complexity and Cognitive Processes

The predicted clusters reveal a continued interest in semantic complexity and cognitive processes. PT Cluster 0 focuses on “polysemy,” “homonymy,” and “lexical ambiguity,” indicating ongoing exploration into how metonymy contributes to semantic richness and ambiguity in language. The inclusion of “construction” and “construal” suggests a focus on the cognitive processes involved in constructing and interpreting metonymic meanings, reflecting the foundational role of cognitive linguistics in metonymy research.

2) Developmental and Conceptual Applications

PT Cluster 1 highlights the application of metonymy in developmental and conceptual contexts. The presence of keywords such as “figurative language,” “language development,” and “word-formation” points to a focus on how metonymy is acquired and utilized across different stages of language development. The mention of “autism spectrum disorder” suggests an interest in how metonymic processes may differ in neurodiverse populations, while “advertising” and “conceptualization” indicate an exploration of metonymy's role in shaping concepts and influencing communication strategies.

3) Rhetorical and Persuasive Dimensions

The rhetorical and persuasive dimensions of metonymy are emphasized in PT Cluster 2, which includes keywords like “persuasion,” “blending,” and “euphemism.” This cluster suggests a focus on the strategic use of metonymy in achieving persuasive communication goals, highlighting its rhetorical power in various discourses. The inclusion of “high-level metonymy” and “conceptual blending” underscores the complex cognitive operations involved in crafting persuasive messages.

4) Social and Cultural Contexts

PT Cluster 3 underscores the relevance of metonymy in social and cultural contexts. Keywords such as “corpus,” “semantic change,” “rhetoric,” “gender,” and “race” indicate a focus on how metonymy reflects and influences social dynamics and cultural narratives. This cluster suggests that metonymy research will continue to engage with issues of identity, power, and social change, leveraging corpus methodologies to analyze large datasets and uncover patterns in metonymic usage.

5) Multimodal and Iconic Aspects

The predicted topics also highlight the expansion of metonymy research into multimodal and iconic domains. PT Cluster 6 includes terms like “metaphor,” “multimodality,” “creativity,” and “visual metaphor,” pointing to an interest in how metonymy and metaphor interact across different communicative modes. This reflects a trend toward integrating visual and multimodal analysis into traditional linguistic studies, examining how metonymic expressions function in visual art, film, and digital media. PT Cluster 4 emphasizes “discourse,” “iconicity,” and “cognitive semantics,” indicating a focus on the interaction between metonymy and discourse, particularly in its iconic and indexical dimensions. This cluster suggests that researchers will explore how metonymic relationships are visually and iconically represented, enhancing our understanding of metonymy's role in creating meaning across different semiotic landscapes.

6) Core Cognitive and Emotional Themes

PT Cluster 5 focuses on core cognitive and emotional themes, with keywords such as “metonymy,” “conceptual metonymy,” “cognitive linguistics,” and “emotion.” This cluster highlights the central role of cognitive and emotional processes in metonymy research, emphasizing the foundational aspects of metonymy that continue to drive inquiry into its cognitive underpinnings and emotional impacts.

### 4.3 Analysis of the evolution of predicted topics

This section compares the relationship between the predicted topics (PT) and the original topics (OT) to understand how metonymy research is expected to evolve. The predicted topics result from the recombination and splitting of keyword groups from the original topics. By analyzing these relationships, we can identify significantly changed predicted topics, which may represent newly emerging scientific topics within the field. The degree of change in predicted topics is measured by the overlap of keyword groups between the predicted and original topics. Overlap is defined as complete lexical matches or semantic equivalence, with the degree of overlap calculated as the ratio of overlapping words to the total number of words in the theme. A threshold of 0.75 is used to differentiate between insignificant and significant changes. Predicted topics with an overlap >0.75 are considered to have insignificant changes, while those with an overlap < 0.75 are seen as significantly changed and are potentially aggregates of multiple original topics.

**Figure 9:**
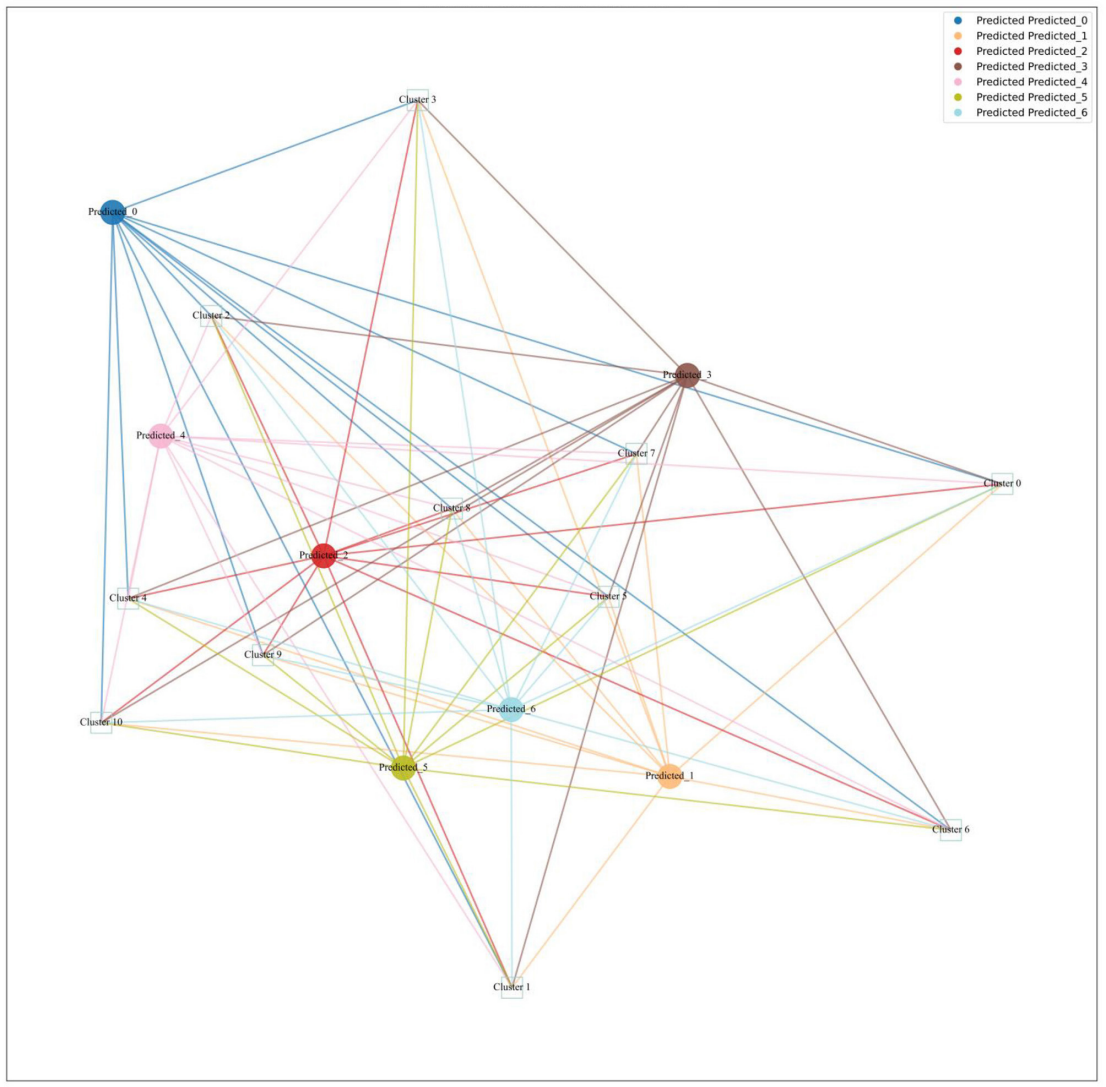
Mapping of predicted topic to original topic.

Figure 9 illustrates the relationships between the original topics and the predicted topics. Each circle represents a Predicted Topic (PT), while each square represents an Original Topic (OT). The lines connecting the circles (PTs) to the squares (OTs) indicate the relationship between specific publication topics and the broader research themes. The proximity of the lines reflects the strength or closeness of the relationship between the PT and OT. Shorter lines suggest a stronger or more direct connection, while longer lines indicate a weaker or more distant relationship.

1) Predicted Topic with Insignificant Changes

PT Cluster 0 aligns with OT Cluster 2 from the original topics, emphasizing semantic complexity through a focus on “homonymy,” “polysemy,” and “lexical ambiguity.” This suggests a sustained interest in exploring how metonymy contributes to semantic richness and complex language construction, reflecting a stable and enduring area of research. Similarly, PT Cluster 1 overlaps with OT Cluster 6, maintaining a focus on language development, conceptualization, and the role of metonymy in neurodiverse contexts such as autism spectrum disorder. This indicates ongoing research into how metonymy aids language acquisition and conceptual growth, highlighting its importance in cognitive and educational perspectives. Meanwhile, PT Cluster 2 aligns with OT Cluster 5, continuing to explore the persuasive uses of metonymy, including “high-level metonymy,” “conceptual blending,” and “persuasion.” This reflects a consistent interest in the rhetorical and strategic applications of metonymy in communication, emphasizing its role in shaping discourse and cognitive blending processes. Lastly, PT Cluster 3 shows strong alignment with OT Cluster 0, focusing on empirical approaches using corpus methodologies to analyze metonymy's role in social and cultural contexts. The continued emphasis on “rhetoric,” “conceptual integration,” and “corpus” indicates a stable research interest in understanding how metonymy influences societal dynamics and cultural narratives, leveraging corpus-based analyses to uncover patterns and variations in metonymic expressions across large datasets. Overall, these themes demonstrate the ongoing relevance and impact of established research areas within metonymy, providing a solid foundation for continued exploration and deeper insights into its role in human communication.

2) Predicted Topic with Significant Changes

The significantly changed predicted topics reveal a dynamic shift in metonymy research, reflecting the merging and recombination of elements from multiple original topics. These predicted topics represent potential new directions and highlight the evolving landscape of metonymy studies. PT Cluster 4 primarily originates from OT Cluster 4, which initially focused on the emotional and iconic dimensions of metonymy. This cluster integrates insights from OT Cluster 3 (Semantics and Corpus Linguistics) and OT Cluster 10 (Visual Metaphor and Political Cartoons), reflecting a new focus on “discourse,” “iconicity,” and “cognitive semantics.” This evolution signifies an interest in exploring how metonymic relationships are represented in discourse, particularly their iconic and indexical dimensions, suggesting a shift toward understanding metonymy within broader communicative and semiotic contexts. PT Cluster 5 emerges from OT Cluster 8 (Multimodal Metonymy in Picture Books) and Cluster 10 (Visual Metaphor and Political Cartoons), with contributions from OT Cluster 5 (Persuasion and Political Communication). This cluster emphasizes “conceptual metonymy,” “visual metonymy,” and emotional processes, building on the original topics' focus on cognitive and visual aspects. The predicted theme represents an expansion toward examining the interplay between cognitive processes and emotional impacts, highlighting a more nuanced understanding of metonymy's role in human cognition and communication. PT Cluster 6 is significantly influenced by OT Cluster 10 and OT Cluster 8, with input from Cluster 9 (Construction Grammar and Analogy). This cluster focuses on “creativity,” “metaphor,” and “multimodal metaphor,” indicating a shift toward integrating multimodal and metaphoric analyses. This evolution reflects a broader interest in exploring how metonymy and metaphor interact across different media, emphasizing creativity and visual communication, and highlighting the trend toward studying metonymy in diverse communicative modes. PT Cluster 3 primarily draws from Cluster 0 (Cognitive Linguistics and Conceptual Metonymy), with additional influences from OT Cluster 3 and OT Cluster 9. It highlights the use of corpus methodologies to analyze metonymy in social contexts, focusing on “rhetoric,” “gender,” “race,” and other social constructs. This shift reflects an emerging interest in how metonymy influences societal dynamics and cultural narratives, leveraging corpus methodologies to provide insights into metonymic expressions across large datasets. Together, these significantly changed predicted topics illustrate the expanding and evolving nature of metonymy research, suggesting a vibrant and interdisciplinary future for the field.

## 5 Conclusion

This study offers a comprehensive analysis of metonymy research, specifically focusing on articles published from 2000 to 2023, identifying key trends and evolving themes within the field. Using bibliometric analysis and clustering techniques, we explored established and emerging areas of metonymy research, highlighting the dynamic interplay between cognitive, semantic, and multimodal dimensions.

### 5.1 Key findings

Our analysis reveals that cognitive and conceptual linguistics remain foundational to metonymy research, with a strong emphasis on understanding the cognitive mechanisms driving the use and interpretation of figurative language. This is evidenced by the consistent presence of keywords such as “metonymy,” “metaphor,” and “cognitive linguistics” across multiple clusters. These findings align with the work of Barcelona (2000) and Kövecses (2002), who argue that metonymy and metaphor often interact in particular linguistic behaviors. These findings reflect the sustained interest in the cognitive processes underlying metonymic expressions and highlight how metonymy research often appears alongside metaphor studies or as a complementary area of inquiry. The close relationship between these two figurative devices indicates their intertwined nature in understanding language and thought, suggesting that they are frequently studied together to provide a more comprehensive picture of figurative language, reinforcing the claims made by Radden and Kövecses (2007) that their overlapping cognitive bases provide an integrated framework for analyzing figurative language. This also supports the possibility of a unified theoretical framework encompassing metonymy, metaphor, and other figures of speech, as proposed by Ruiz de Mendoza (2020).

The study also points to the importance of semantic and pragmatic dimensions, focusing on how metonymy functions at the level of meaning and use. This trend builds distinguished pragmatic types of metonymies (see Panther and Thornburg 1998). The exploration of semantic complexity and lexical ambiguity, particularly in PT Cluster 0, indicates a still ongoing interest in how metonymy contributes to semantic richness and ambiguity in language. A notable trend in metonymy research is the expansion into multimodal and visual contexts, as highlighted in PT Cluster 6. This reflects a growing interest in how metonymy operates across different communicative modes, including visual art (Uno et al., 2019), film (Feng, 2017), and digital media (Bolognesi et al., 2019). The integration of linguistic analysis with modern technological tools suggests a recognition of the importance of studying metonymy in visual and digital contexts. Additionally, the study highlights the application of metonymy theory in social and cultural contexts, exploring its role in shaping societal discourses and identity constructions. PT Cluster 3 emphasizes the relevance of metonymy in social and cultural domains, a theme that resonates the work of Kövecses (2005, 2006), focusing on how metonymy reflects and influences societal dynamics and cultural narratives.

Finally, the predicted topics indicate potential future directions for metonymy research, emphasizing the evolving landscape of the field. Significantly changed themes, such as those in PT Clusters 4 and 5, suggest new areas of inquiry, including the interaction between metonymy and discourse and the exploration of cognitive and emotional processes.

### 5.2 Strengths and limitations

Our methodological approach, which integrates bibliometric analysis with clustering techniques, proves to be highly effective in mapping the intellectual structure of metonymy research. The use of co-citation and co-word analysis, combined with advanced clustering algorithms such as k-means, allows for the identification of distinct research themes and the tracking of their evolution over time. This approach provides a robust framework for exploring large datasets, enabling a detailed understanding of the field's development and the interconnections between various research areas. Moreover, the predictive modeling using ARIMA and other forecasting methods offers valuable insights into potential future trends, guiding researchers in identifying emerging areas of interest.

However, it is important to acknowledge the limitations of this methodological approach. The analysis focused exclusively on articles indexed in the SSCI, excluding books published during the same period (2000–2023), which may also contain valuable insights into metonymy research. While bibliometric and clustering techniques are powerful tools for analyzing research trends, they rely heavily on the quality and scope of the underlying data. The selection of keywords, the accuracy of citation databases, and the inherent biases in publication practices can all influence the results. Additionally, the focus on quantitative analysis may overlook nuanced qualitative aspects of metonymy research, such as the depth of theoretical discussions or the subtleties of interdisciplinary integration. Future research could benefit from combining these quantitative methods with qualitative analyses to provide a more comprehensive understanding of the field.

### 5.3 Implications for future research

Overall, this analysis provides a roadmap for future studies, encouraging continued exploration and discovery within the diverse and multifaceted realm of metonymy. By building on the insights gained from this study, researchers can further expand the boundaries of metonymy research, ensuring its continued relevance and contribution to linguistic, cognitive, and social sciences. To provide more precise guidance for future research, it would be beneficial to explore specific applications of metonymy in digital communication platforms, assess the impact of cultural differences on metonymic usage, and develop innovative computational models to analyze large-scale data sets. Additionally, future studies could employ mixed-method approaches, combining ethnographic methods with quantitative data analysis, to gain deeper insights into the usage of metonymy across different languages and cultures.

## Data Availability

The raw data supporting the conclusions of this article will be made available by the authors, without undue reservation.
